# Cytomegalovirus (CMV)-Specific Perforin and Granzyme B ELISPOT Assays Detect Reactivation of CMV Infection in Inflammatory Bowel Disease 

**DOI:** 10.3390/cells1020035

**Published:** 2012-04-23

**Authors:** Tobias M. Nowacki, Dominik Bettenworth, Matthias Ross, Jan Heidemann, Paul V. Lehmann, Andreas Lügering

**Affiliations:** 1 Department of Medicine B, University of Münster, Münster 48149, Germany; E-Mails: tobias.nowacki@ukmuenster.de (T.M.N.); dominik.bettenworth@ukmuenster.de (D.B.); matthias.ross@ukmuenster.de (M.R.); jan.heidemann@ukmuenster.de (J.H.); 2 Cellular Technology Limited, Shaker Heights, OH 44122-5350, USA; E-Mail: paul.lehmann@immunospot.com; 3 Medical Care Center Portal 10, Münster 48155, Germany

**Keywords:** cytomegalovirus infection, CMV, ELISPOT, immunomonitoring, inflammatory bowel disease

## Abstract

The role of cytomegalovirus (CMV) infection in the pathogenesis and exacerbation of Inflammatory Bowel Disease (IBD) has been unresolved. Typically, the CMV genome remains dormant in infected cells, but a breakdown of immune surveillance can lead to re-activation of viral replication in the gut mucosa, which is not necessarily associated with viremia or changes in antibody titers. We hypothesized that the detection of CMV-specific CD8 effector T cells should permit the distinction between dormant and active CMV infection. As CD8 effector T cells, unlike memory CD8 T cells, have perforin (PFN) and granzyme B (GzB) preformed in their cytoplasmic granules, we employed single cell resolution ELISPOT assays to measure the CMV antigen-triggered release of these molecules by CD8 T cells isolated from subjects with IBD, and age-matched healthy controls. The frequencies of CMV-specific (GzB) and PFN-producing CD8 T cells were increased in IBD patients compared to healthy controls. Furthermore, the increased CMV reactivity was associated with active IBD disease and with longer disease duration. Notably, PCR on serum frequently failed to detect CMV DNA during flares. The data show that during active IBD there is a flare of CD8 T cell activity against CMV in a substantial proportion of IBD patients, suggesting CMV reactivation that serum PCR does not detect. While it remains open whether CMV reactivation is a cause or consequence of IBD, our data suggest that monitoring CMV antigen-specific effector CD8 T cells with GzB and PFN ELISPOT analysis can provide novel insights into the role of CMV infection in IBD. Additionally, our data have implications for the fields of transplantation, HIV, cancer, and autoimmune diseases, in all of which patient care critically depends on sensitive and reliable detection of a reactivation of CMV infection.

## Abbreviations

CMV: cytomegalovirus
GzB: granzyme B
PFN: perforin
IBD: inflammatory bowel disease

## 1. Introduction

The etiology of Crohn’s disease and ulcerative colitis remains unknown. Various reports suggest that viral infections, CMV in particular, are associated with the onset and aggravation of IBD [1,2,3,4]. It is unclear whether these infections occur as cause or consequence of IBD, or as a complication of its treatment; in addition, the flare of the infections in itself represents a complication for disease management. The prevalence of CMV infection in IBD patients has been estimated to range from 21% to 34% in patients suffering from severe ulcerative colitis and from 33% to 36% in patients with steroid refractory ulcerative colitis [3,5,6,7]. 

CMV, like all related herpes viruses, typically remains latent within the body over long periods of time, but can give rise to renewed viral replication also leading to a flare of the anti-CMV T cell response. Viral replication is primarily controlled by CD4 and CD8 T cells. Presently, diagnostic algorithms allowing for a reliable distinction between latent and active infection are missing. In the case of IBD, reactivation or *de novo* infection can worsen colitis and cause steroid refractory disease whereas in other situations evidence of a viral infection might be of little pathogenic relevance [4,8,9]. The challenge to distinguish between latent and active infection is further complicated by the fact that most tissues in the body can be infected whereby immune surveillance and viral replication in the different organs follow fundamentally different rules. Moreover, a multitude of techniques have been proposed to monitor CMV infections, among which are endoscopy, histology, serology, viral culture, antigen testing and DNA testing [10,11,12]. Yet, none seems sufficient enough to reflect CMV activity in general, and in IBD in particular. For example, the detection of the CMV genome in blood or intestinal tissue was associated with a short duration of IBD but not with the severity of the disease, its activity at colonoscopy or the development of pancolitis [13]. 

Immune-mediated therapies for IBD are primarily immunosuppressive or immunomodulatory in nature. However, immunosuppressive or immune modulating drugs render patients susceptible to the reactivation of latent viral infections such as CMV [14]. Without the ability to reliably monitor the reactivation of the CMV infection, clinicians cannot identify patients at risk to develop complications of such, and cannot provide rational treatment.

Recently, GzB and PFN ELISPOT assays have been introduced that allow for the identification of CD8 effector T cells that had been actively involved in immune surveillance *in vivo* [15,16]. CD8 T cells play a crucial role in the control of viral infections [17,18,19]. Before the first encounter of “their” antigen, CD8 T cells are naïve, occur in low clonal sizes, do not express PFN and GzB, and are not capable of killing [20]. Infection or vaccination triggers the specific naïve CD8 T cells to engage in an immune response: these CD8 T cells undergo clonal expansion, start synthesizing and storing GzB and PFN, and acquire the ability to kill [21]. They can also secrete IFN-γ, TNF, and other effector molecules. Such recently activated CD8 T cells are called effector cells. CD8 effector T cells are short lived. As soon as the virus is cleared, or if a virus persists, as soon as viral replication stops, they become quiescent memory cells that do not express PFN and GzB anymore, and temporarily lose their ability to kill. Upon renewed antigen encounter, the memory cells are capable of secreting cytokines such as IFN-γ; however it takes about 3 days for them to replenish their granules with PFN and GzB and to reacquire the ability to kill. In this reactivated state they are referred to as effector memory cells [22,23,24]. CD8 effector and effector memory T cells kill target cells utilizing PFN and GzB: the effector cells release these molecules towards the target cell [25,26], PFN punches holes into the cell membrane through which GzB penetrates to induce apoptosis via activation of caspase pathways [27,28,29]. CD8 T cells can also kill utilizing Fas–FasL interactions [30,31]. In the absence of continued antigen stimulation (in the case of CMV, in the absence of active viral protein synthesis), also CD8 effector memory T cells become quiescent, acquire a resting phenotype, and within about 30 days lose PFN and GzB. They continue to be able to secrete IFN-γ, however. Such resting CD8 cells are called central memory T cells [32]. 

Therefore, the ability to instantaneously engage into PFN and GzB secretion is a characteristic of CD8 effector and effector memory T cells that have had antigen exposure *in vivo* less than 30 days ago. In contrast, CD8 memory T cells that encountered antigen in the distant past are not capable of instantaneous PFN and GzB secretion, but can re-acquire it within 3 days [22]. Therefore, PFN and GzB ELISPOT assays of 24 h duration detect only antigen-specific CD8 effector/effector memory T cells permitting the identification of antigens that are being actively targeted by CD8 T cells *in vivo* [15,16]. In this study, we tested the hypothesis that CMV is actively recognized in IBD by CD8 T cells. 

## 2. Results

### 2.1. Increased Frequencies of Cytomegalovirus CMV-Reactive T Cells Producing IFN-γ, Granzyme B (GzB), and Perforin (PFN) in Inflammatory Bowel Disease (IBD) Patients

**Figure 1:**
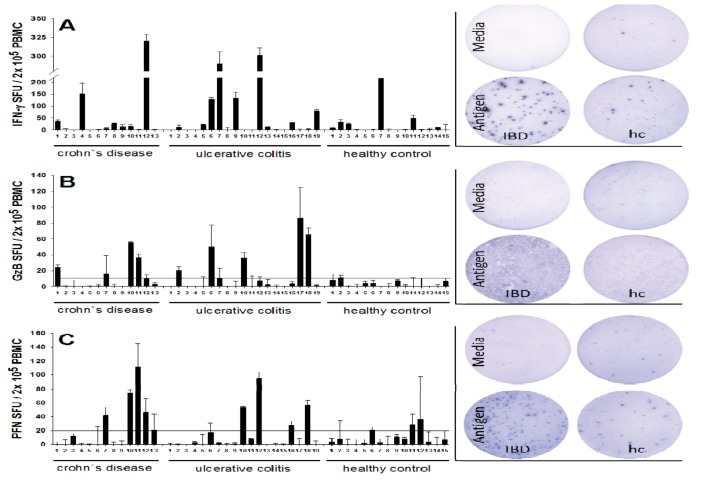
Detection of Cytomegalovirus (CMV)-reactive effector T cells in Inflammatory Bowel Disease (IBD) patients and healthy controls. Peripheral blood mononuclear cells (PBMC) from 32 patients with IBD (ulcerative colitis (UC) or Crohn’s disease (CD)) and from 15 healthy control donors (hc) were plated at 200,000 cells per well and cultured with a CMV peptide pool containing 5 immunodominant peptides of CMV for 24 h, and the peptide-induced production of IFN-γ (panel A), GzB (panel B) and perforin PFN (panel C) was measured in duplicate wells in an ELISPOT assays specific for the respective analyte. Each column represents the test result for a single donor with the mean spot count per well shown (±SD). The applied cutoff is indicated as a dotted line in panel B and C. Representative images are shown for each analyte.

We tested in freshly isolated peripheral blood mononuclear cells (PBMC) the frequencies of CMV antigen-specific CD8 T cells that qualify as effector/effector memory cells by virtue of their ability to secrete GzB and PFN, as only CD8 T cells that had been recently activated *in vivo* secrete these molecules when challenged *in vitro* in an ELISPOT assay of 24 h duration. PBMC were isolated from 32 IBD patients and 15 healthy controls. The cells were plated at 200,000 cells per well, and challenged with a CMV peptide pool that contained 5 immunodominant peptides of the virus. An ELISPOT assay of 24 h duration was performed to measure numbers of cells that engage in peptide-induced production of IFN-γ, GzB and PFN. In previous studies we [15,16] and others [33] established that these CMV peptides activate CD8 cells, and that the peptide-induced IFN-γ, GzB, and PFN are CD8 cell derived. The results are summarized in Figure 1. A positive release of GzB (>10 SFU ± SD) and/or PFN (>20 SFU ± SD) was detected in 12 of 32 (37.5%) of IBD patients, whereas none of the control samples showed a significant release of GzB or PFN after stimulation (p < 0.05). The frequency of GzB and PFN producing CMV peptide-specific cells reached up to 90 and 100 with means 12 and 18, respectively, within the 200,000 PBMC tested. In the healthy controls, in contrast, the maximal numbers of GzB- and PFN- producing CMV-specific T cells was less than 20 and 40, with means 3 and 9 respectively, within the 200,000 PBMC tested. Also, the frequencies of CMV peptide-specific IFN-γ producing cells were higher in the IBD patients, reaching a maximum at 320 per 200,000 PBMC, and a mean of 50, relative to the controls (maximal frequency 210, and a mean of 23 per 200,000 PBMC). CMV-reactive T cells were observed in patients suffering from ulcerative colitis (n = 7) as well as Crohn’s disease (n = 5). A proportional distribution was found between male and female patients (not shown).

### 2.2. Increased Per Cell IFN-γ Productivity of CMV-Reactive T Cells in IBD Patients

**Figure 2:**
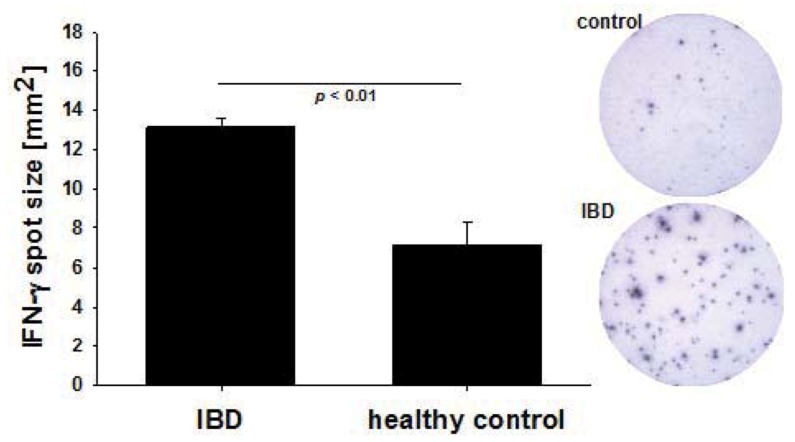
IFN- γ spot size measurements. IFN- γ spot size – a reflection of the amount of cytokine released by each cell – was measured comparing IBD patients, who tested positive for *ex vivo* GzB and PFN secretion and healthy controls. Representative images are shown. Statistical significance was evaluated by Student’s t-test.

In addition to the frequency, the mean IFN-γ spot size was also significantly higher in the IBD patients who tested positive for GzB and PFN secretion as compared to healthy controls (13.12 ± 0.45 sqmm *vs*. 7.11 ± 1.13 sqmm, p < 0.05) (Figure 2). IFN-γ is secreted by both resting memory T cells and recently-activated T cells. Increased per cell IFN-γ productivity that is reflected in ELISPOTs of increased size, is, like PFN and GzB secretion, a characteristic of CD8 T cells that had been recently activated *in vivo* [34]. In contrast to IFN-γ, GzB and PFN are not produced by resting CD8 T cells, and activated CD8 T cells produce these analytes for a couple of weeks after activation. Consistent with this notion, we have found GzB and PFN producing CMV-specific CD8 cells in a subset of IBD patients, but not in healthy controls. Unlike for IFN-γ, therefore, recall antigen-induced GzB and PFN spots sized cannot be compared. Therefore, four independent parameters of CD8 T cell immunity suggest a recent anti-CMV immune activation in IBD patients: namely increased clonal sizes, secretion of GzB, of PFN, and increased per cell IFN-γ productivity. 

### 2.3. CMV-Specific T Cell Activity *vs*. the Clinical Activity of IBD

**Figure 3:**
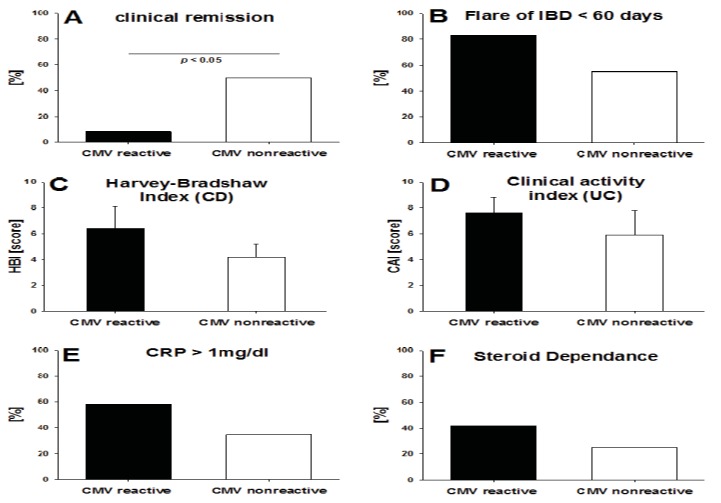
Detection of CMV reactive T effector cells in relation to disease activity. Each panel specifies a clinical criterion for ulcerative colitis (UC) and/or Crohn`s disease (CD). The percentages of GzB/PFN responsive *vs*. non responsive subjects with IBD (represented by the solid and open bars, respectively) are shown for each criterion. Also, disease activity indices are depicted for patients with CD and UC. Reactive IBD subjects were defined as individuals in whose PBMC the CMV peptide pool elicited spot counts of ≥10 GzB-SFU ± SD, ≥20 PFN-SFU ± SD (background subtracted) respectively. The Mann–Whitney rank sum test was used to evaluate statistical significance of differences in disease activity indices (panel C and D). For all other clinical criteria Fisher’s exact test was used.

While the number of IBD subjects studied was not high enough to obtain definitive results regarding how T cell reactivity to CMV reflects on the clinical course of IBD, initial trends have become apparent. For the 32 IBD subjects studied, we compared patients with a positive GzB/PFN response and non-responders with regard to disease activity indices, as well as C-reactive protein serum levels, steroid medication and the presence of disease flares (Figure 3). Only 1 of 12 IBD patients that displayed strong signs of recent T cell activation against CMV was in clinical remission (HBI ≤ 4 or CAI ≤ 3) compared to 12 of 20 patients in the group that showed low level of T cell activation to CMV (p < 0.01) (Figure 3A). An acute flare-up of disease had occurred in 10 of 12 (83%) patients with signs of recent T cell activation to CMV *vs*. in 11 of 20 (55%) IBD patients who displayed only resting memory T cell activity to CMV. In both cases, our definition was that the flare-up needed to occur within the last 60 days prior to the T cell testing (Figure 3B). At the time of visiting, patients suffering from Crohn’s disease as well as ulcerative colitis both exhibited an increased disease activity score when GzB and PFN reactive for CMV peptides compared to non-reactive patients (Harvey Bradshaw index 6.4 ± 3.78 *vs*. 4.1 ± 3.45; Colitis activity index 7.6 ± 3.13 *vs*. 5.9 ± 5.7) (Figure 3C–D). In addition, 58% of the patients with a positive CRP level (CRP > 1.0 mg/dl) showed evidence of recent T cell activation, while only 35% of the non-responsive patients had CRP levels exceeding 1.0 mg/dL. (Figure 3E). Also, the proportion of patients requiring a daily steroid treatment of ≥10 mg prednisone for longer than 4 weeks prior to analysis for CMV reactivity was higher in CMV reactive patients (42% *vs*. 25%) (Figure 3F). Because the IBD patients were under variable therapies, receiving different immunomodulators (azathioprine, mercaptopurine, methotrexate) as well as anti-TNF agents, we did not have sufficient subjects in our study to analyze the impact of the respective therapy on the T cell reactivity to CMV. Laboratory data such as leukocyte- and thrombocyte count, GPT, and creatinine were not different between the GzB/PFN responsive and the non-responsive IBD patients. 

### 2.4. Detection of T Cell Activation by CMV *vs*. Viral Replication Measured by PCR

None of the IBD patients showed a positive result for CMV DNA at the time tested for ELISPOT CMV-reactivity (Table 1). Three patients had a known history of a positive CMV-PCR multiple months before the study but did not display strong GzB or PFN responses: this observation is in line with the notion that the PFN/GzB positive state of effector/effector memory CD8 T cells last for about one month. Overall, strong signs of recent T cell activation in the absence of detecting an increase in viral load in the blood by PCR suggest that the T cell test is more sensitive in detecting reactivation of the viral infection. 

**Table 1:** Detection of CMV viral replication measured by PCR. When blood was obtained from IBD patients (ulcerative colitis (UC) or Crohn’s disease (CD)) for PBMC isolation for ELISPOT CMV-reactivity analysis, CMV DNA was also measured by PCR in blood samples.

CMV DNA PCR replication measurements			
	CMV DNA PCR [copies/mL]		
	at ELISPOT testing	1 month prior	≥ 2 months prior
IBD patient			
all CD, all UC except 8,14,19	neg.	not tested	not tested
UC 8	neg.	2270	not tested
UC 14	neg.	not tested	1892
UC 19	neg.	5403	31000

### 2.5. Disease Duration in IBD Patients with High CMV-Reactivity

**Figure 4:**
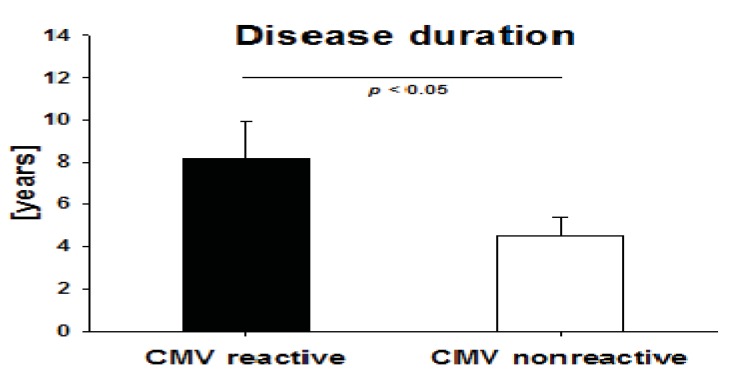
Disease duration in CMV reactive patients. Mean duration of Inflammatory Bowel Disease (Crohn’s disease and Ulcerative colitis) was established for patients who tested positive for CMV reactivity in GzB or PFN ELISPOT assays *vs*. nonresponders. Statistical significance was evaluated by Mann–Whitney rank sum test.

We tested whether IBD duration influences the incidence of active T cell immunity against CMV in IBD patients. We therefore compared both groups (patients with signs of recent T cell activation against CMV, denominated by the detection of GzB and/or PFN *vs*. patients with no GzB/PFN response) regarding the time that had elapsed since the first definitive diagnosis of IBD. CMV-reactive patients had a significantly longer disease duration when compared to non-CMV reactive patients (8.16 ± 1.7 years *vs*. 4.5 ± 0.86 years, p < 0.05) (Figure 4).

## 3. Discussion

In this study, we show for the first time that a significant fraction of IBD patients exhibit a recently activated immune response to CMV, possibly drawing attention to the relevance of this herpes virus infection in the context of IBD. CMV-specific T cells were found to be in an activated state in a significant number of patients suffering from Crohn’s disease as well as ulcerative colitis, in particular during flare-ups of the disease, whereas no anti-CMV effector T cell activity was found in healthy controls. 

The difference between the groups with respect to responsiveness *vs*. non-responsiveness was clear cut, suggesting that follow-up studies should be warranted. In contrast to the dichotomous positive/negative evaluation of the data, the number of subjects studied was not high enough to permit reliable statistical evaluation based on the mean number of spots in each group. As opposed to the binary positive/negative assessment, systematic future studies in larger cohorts of patients and controls will be required to provide a definitive answer to the question of whether the observed differences in a subgroup of IBD patients apply to IBD patients in general. A comparison of disease activity indices in GzB/PFN responsive and non-responsive IBD patients demonstrated that GzB/PFN responsive patients were seven times less likely to be in clinical remission compared to non-responsive patients. In contrast, clinical IBD activity was not correlated with the detection of CMV DNA in the serum as none of the patients tested positive for CMV DNA by PCR. This finding can be reconciled with the very nature of immune surveillance by T cells. CMV infects many tissues of the body, including the gut mucosa, where it can stay latent for prolonged time periods. In the context of T cell immunity, latency means that no viral proteins are actively biosynthesized. While viral DNA, and possibly even viral proteins synthesized in the past are present in infected cells, it takes *de novo* intracellular protein synthesis to load viral peptides onto HLA-class I molecules (HLA-A, B and C) and to display these to T cells on the surface of the infected cells. Thus, as soon as viral protein synthesis initiates in an infected tissue, such as the gut mucosa, viral peptides become presented to T cells on the infected cells. It takes only a few viral peptide-loaded HLA-class I molecules to activate a CD8 T cell. Therefore, the T cell system responds to trace amounts of antigen, in any organ: CD8 T cells actively survey all tissues of the body migrating from organ to organ seeking for “their” antigen. Since T cells utilize the bloodstream for their dissemination in the organism and recirculation, activated T cells can be readily detected in the blood even if T cell activation occurred e.g., in the mucosal tissue of the gut. In contrast, viremia resulting from massive viral replication is required for viral DNA to be detectable in the serum. Moreover, for detection by PCR in the serum, virions need to be present in the blood in spite of the presence of neutralizing antibodies that all CMV-infected individuals have. These antibodies precipitate virions in the infected tissue, and virions that reach the blood will be coated with antibody and trapped in immune complexes that are rapidly cleared from circulation in the reticulo-endothelial system, primarily the lungs, liver and spleen. Therefore, the detection of active CMV replication via the emergence of activated virus-specific T cells in the blood should be more sensitive than virion detection in serum by PCR. To our knowledge, this is the first report that provides experimental evidence in support of this notion. Detecting CMV–specific activated T cells, therefore, could gain substantial clinical significance for patient care managing complications of CMV infection. By extrapolation, GzB and PFN ELISPOT assays should also permit the detection of a reactivation of other viral infections, or re-infections. Hereby, the use of readily available unseparated PBMC (as pertinent for clinical immune diagnostic) seems justified, since previous studies have confirmed the CD 8 T cell origin of the measured cytokines when using HLA Class I restricted T cell epitopes for antigenic stimulation [15,16,33].

Serological tools (CMV-specific IgM and IgG antibodies) are very sensitive and specific in detecting whether an individual has been infected with CMV, but do not distinguish between a latent state and re-activation of the infection. After re-activation, antibody titers change slowly over several weeks to months and such changes are not necessarily related to an active immune response. CMV antigens (late structural protein pp65), like CMV DNA, can be detected in serum and other body fluids. However, for the reasons discussed above, previous studies suggested that the absence of viral DNA might not be indicative of a CMV unrelated disease event [35]. Additionally, positive testing for CMV DNA or pp65 Ag might also not reflect an active immunologic process within the intestinal mucosa. 

The detection of CMV in the inflamed colonic mucosa is at present the gold standard for identifying CMV-related intestinal disease. However, the detection rate is limited even in the case of an otherwise proven CMV disease, even if CMV-directed antibodies or DNA hybridization methods are used. More importantly, CMV detection in the mucosa does not necessarily reflect active biosynthesis of the virus, and therefore the display of viral peptides to the T cell system resulting in an active immune response. In addition, this method is dependent on biopsy material that, unlike PBMC, is not readily available for routine clinical monitoring. 

Our data suggest a link between active CMV infection and IBD, but leave mechanistic interpretations open. Similarly to other herpes virus-mediated diseases, acute infections are frequently asymptomatic, but once acquired, the virus persists lifelong. In IBD, the reactivation of CMV infection is thought to cause severe colitis, particularly in patients with ulcerative colitis that are treated with immunosuppressive agents. However, in these cases it is hard to determine whether CMV is a non-pathogenic bystander or if the virus is triggering the onset of the disease, and if the therapeutic strategy should be adapted to this finding. Indeed, Eyre-Brook demonstrated that antiviral treatment only marginally alters the course of IBD [36]. CMV might also spontaneously be cleared over time as shown by Matsuoka *et al*. [37]. However, other studies provided evidence that it is crucial to confirm an active ongoing infection by CMV to deflect a therapeutic consequence [38]. Some authors have suggested a crucial role of the inflamed mucosa in inducing and sustaining CMV reactivation as epithelial cells can serve as permissive hosts for CMV during inflammatory responses. This could possibly create a permissive environment for CMV reactivation and replication which in turn enhances the chance of chronic viral infection, resulting in an increased proportion of CMV infections over time [39]. Yet, the data on the correlation between duration of disease and CMV infection are conflicting. While some studies report shorter disease duration in CMV positive patients, others saw a trend towards a longer duration [13,40]. This disparity is most likely due to the different diagnostic approaches towards defining a CMV infection. As we pointed out before, none of the CMV-reactive patients was positive for serum CMV DNA at the time tested. 

IBD patients are usually immunosuppressed due to a number of reasons, such as poor nutrition, possible NK cell impairment or immunosuppressive treatment. Together with CMV tropism for sites of inflammation, these factors leave IBD patients at increased risk of active CMV infection. CMV infects areas of active IBD and causes colonic injury in most cases. As viral multiplication is most likely a localized phenomenon strictly occurring within the inflamed mucosa, it is conceivable that mucosal CMV replication triggers acute and fulminant courses of disease, while in other cases the prolonged history of inflammation favors the acquisition of CMV infection in a long course of disease (Schema 1). Our finding that CMV responsiveness is associated with longer disease duration as well as greater disease activity suggests that mucosal injury over a long course of disease might establish a permissive environment for CMV acquisition and replication. However there do seem to be situations in which CMV infection and replication leads to enhanced tissue damage, causing a breakdown of mucosal barrier functions and subsequent development or exacerbation of acute and fulminant courses of IBD.

**Schema 1:**
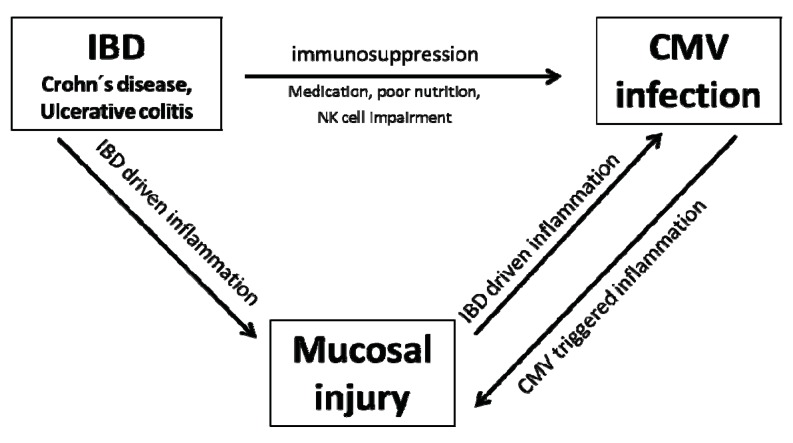
CMV infection an IBD.

## 3. Experimental Section-Materials and Methods

### 3.1. Subjects, Cell Separation

Peripheral blood was obtained from 32 IBD patients routinely visiting the outpatient IBD clinic of the University of Münster as well as the Medizinisches Versorgungszentrum Portal 10 after informed consent. Data regarding their disease were recorded, standard laboratory marker (CRP, leukocyte and thrombocyte count, ALT, creatinine) measured and IBD indices (Harvey Bradshaw index, Colitis activity Index; [41,42]) determined. The patient characteristics were as follows: 13 patients with Crohn`s Disease, 19 with Ulcerative Colitis, aged between 20–65 years (mean 37) and a disease duration ranging from 0–21 years (mean 5). 21 were male, 11 females. Extraintestinal manifestations were observed in 19%. All patients were negative for CMV DNA by real-time PCR testing at the time tested for ELISPOT CMV-reactivity. Three patients had a known history of a positive CMV-PCR and subsequent antiviral treatment with ganciclovir multiple months before ELISPOT testing with viral loads reaching from 1800–31,000 copies/mL.

Laboratory parameters were as follows: Creatinine ranged from 0.5–1.2 mg/dL (mean 0.9), Alanin Aminotransferase from 5–81 U/L (mean 21), C-reactive Protein from <0.5–36.7 mg/dL (mean 0.8), Leukocyte count from 3.7–18.0 10^3^/µL (mean 7) and Thrombocyte count from 162–512 10^3^/µL (mean 326.5). 47% of patients were treated with steroids, while 59% received steroids for >3 months and 59% received a steroid dosage of >40 mg (prednisolone equivalent) within the last 12 months. 31% received azathioprine at visit, 28% anti-TNF-α therapies and 16% had received antibiotics in the past 3 months. Endoscopic examinations were not performed. An aged matched control cohort of 15 volunteers was used as reference. All studies were performed under the approval of the Institutional Review Board for Human Investigation at the University Hospital of Münster. 

PBMC were isolated from heparinized blood by standard Ficoll-Hypaque density gradient centrifugation (using Biocoll, Biochrom AG Berlin, Germany). Positivity for HLA-A2 haplotype was assessed by FACS analysis using a FITC-labeled mouse anti human HLA-A2 Antibody (BD Pharmingen, Franklin Lakes, NJ, USA). Cells were subsequently frozen and stored in liquid nitrogen storage until further usage while remaining fully functional as has been previously described [43]. Briefly, PBMC were suspended at a concentration of 2 × 10^7^/mL in 2 mL of freezing medium A (60% FCS, 40% RPMI) at room temperature. An equal volume of freezing medium B (20% DMSO, 80% FCS), was added dropwise. The cell suspension was aliquoted into 1.8 mL cryovials (Greiner Labortechnik, Frickenhausen, Germany), frozen overnight in a −80 °C freezer before transfer to a liquid nitrogen tank for indefinite storage until testing. 

### 3.2. CMV PCR Assay

All CMV PCR assays were performed in the Center for Laboratory Medicine of the University Hospital of Münster that operates under GLP and is accredited to perform routine CMV PCR analysis. Genetic material was extracted from EDTA blood samples using QIAamp MinElute Virus Spin Kit (QIAGEN, Hilden, Germany). An in-house, real-time TaqMan PCR assay with specific primers that amplify a sequence of the Major Immediate-Early Gene of HCMV was used (sequences of the forward and reverse primers: 5’-_2791_AGCGCC GCA TTG AGG A_2806_-3’ and 5’-_2833_CAG ACT CTC AGA GGA TCG GCC_2853_-3’, respectively. Sequence of the probe: 5’-_2808_ATC TGC ATG AAG GTC TTT GCC CAG TAC ATT_2827_-3’). The TaqMan probe was labeled at the 5’ end with 6-carboxyfluorescin and at the 3’ end with 6-carboxytetramethylrhodamine (Applera). PCR amplification was performed using an ABI prism 7700 instrument (Applera) in a total volume of 25 μL with TaqMan Universal PCR master mix 2× (Applera) and each primer at 300 nM and the probe at 200 nM. Cycling conditions were as follows: 2 min at 50 °C, 10 min at 95 °C, and 45 cycles of 95 °C for 15 s and 60 °C for 1 min. A plasmid containing the amplified sequence of AD 169 strain was used as an external standard with amplifications of serial dilutions (range, 10^5^ to 10 copies) allowing for the construction of a standard curve and the quantification of CMV in clinical samples. Results are reported as the number of CMV genome copies/ml of whole blood with a calculated detection threshold of ≥200 copies/mL.

### 3.3. Antigens

A CMV-peptide pool containing five HLA Class I restricted T-cell epitopes from human Cytomegalovirus was obtained from CTL Europe GmbH (Bonn, Germany). Immunodominant HLA Class I peptide determinants for CMV recognized by CD8 T cells have been characterized previously [44,45,46,47]. The chosen epitopes are presented by the most common Caucasian HLA types and have been shown to induce CD8 T cell recall responses in ELISPOT assays in the majority of Caucasians who have been previously exposed to CMV [33]. The following peptides are contained in the pool: NLVPMVATV (HCMV, HLA-A2), SDEEEAIVAYTL (HCMV, HLA-B18), IPSINVHHY (HCMV, HLA-B35), EFFWDANDIY (HCMV, HLA-B44), and TPRVTGGGAM (HCMV, HLA-B7). Additionally, HLA-A2-restricted peptides of CMV (HCMV pp65, peptide 495–503 NLVPMVATV, and HCMV AE 44 VLGPISGHV) were purchased from CTL Europe GmbH (Bonn, Germany) and used to confirm results obtained from peptide pool experiments if the ELISPOT analysis exhibited a marginally positive reaction after stimulation with the peptide pool (n = 5). Only individuals reacting to at least one single peptide were considered positive.

### 3.4. Cell Thawing and ELISPOT Assays

Cells were thawed as previously described [43]. Briefly, cryotubes were placed in 37 °C water until the sample had completely thawed. Cells were transferred into a 50 mL tube containing a 2-fold volume of complete RPMI medium (93% RPMI- 1640, 5% heat-inactivated AB serum, 1% L-glutamine 1% Penicillin–Streptomycin) at room temperature and subsequently washed twice. Interferon-γ, GzB-, and PFN-release was measured by ELISPOT assays as described previously [48]. Briefly, MultiScreen-IP plates (Millipore, Billerica, MA, USA were coated overnight at 4 °C with the cytokine-specific capture antibody diluted in phosphate buffered saline (PBS). Before plating of cells and antigens, the plates were blocked with bovine serum albumin (BSA) (10 g/L in PBS: PBS–BSA) for 1 h and washed 3× with PBS. PBMC were plated in complete RPMI medium at 2 × 10^5^ cells per well (as specified in the table and figure legends). Pre-established optimal antigen concentrations were used for the ELISPOT assay [48]. All peptides were used at 20 µg/mL; phytohemagglutinin (PHA) was used as positive control in all assays and obtained from Sigma (10 µg/mL). Negative control wells contained PBMCs with medium alone. Experiments were performed in duplicate wells for each antigen and cytokine tested.

24 h ELISPOT assays were used for IFN-γ, GzB, and PFN. Thereafter, the plates were washed 3× with PBS, then 3× with PBS–Tween (0.025%), and the detection antibodies (in PBS–BSA–Tween) were added at previously established concentrations. After an overnight incubation at 4 °C, plates were washed 4× with PBS–Tween and Streptavidin-AP (DakoCytomation, 1/1000 dilution) diluted in PBS/BSA/Tween was added to all plates, which were then incubated for 1 h at room temperature. The plates were developed on the same day by using nitroblue tetrazolium 5-bromo-4-chloro-3-indolyl phosphatase substrate. The reaction was stopped by rinsing with distilled water when spots became clearly visible macroscopically (10 to 45 min, dependent on the cytokine) 

The plates were then air-dried overnight before subjecting them to ELISPOT counting using an ImmunoSpot-Analyzer (Cellular Technology Ltd.) specifically designed for the ELISPOT assay. The methods and criteria for the automated counting of spots in the ELISPOT assay have been described previously in detail [15,49,50,51]. Briefly, the ImmunoSpot software uses pattern recognition algorithms to analyze digitized images for the presence of areas in which color density exceeds background. After background noise subtraction, the density distribution is analyzed to identify spots of specific morphology, separating touching and overlapping spots. The measurement of spot-size distribution is also a built-in function of the ImmunoSpot software. Data are recorded as mean spot forming units (SFU) per well of duplicate wells (±SD), *i.e*., the number of spots/well. For the ELISPOT assays, ready-to-use primary and secondary antibodies as well as development solution were obtained from Hölzel Diagnostika GmbH (Cologne, Germany). The following capture-antibodies were used: IFN-γ (B-B1), GzB (GB-11) and PFN (B-E48). The following biotinylated detection antibodies were used: IFN-γ (B-G1), GzB (GB-10) and PFN (B-C48). All Antibodies were used according to the manufacturer’s instructions.

### 3.5. Statistics

Statistical analysis was performed using the Mann-Whitney U (Rank-Sum Test), Student’s *t*-test or Fisher’s exact test where indicated.

## 4. Conclusions

Even though this study was primarily designed to confirm the presence of recently activated anti-CMV immune responses in the context of inflammatory bowel disease by ELISPOT, it also demonstrated that CMV-reactive patients also tend to have a more severe disease with a significantly smaller amount of patients being in remission. Therefore, these data provide a rationale for further exploration in the clinical context. 

Larger studies will be needed to evaluate the association of CMV immune reactivity to e.g., IBD history and phenotype. It also seems reasonable to analyze CMV reactivity after induction of active therapies to identify drugs that have a risk of reactivation of CMV infection as shown previously for azathioprine. Finally, the hypotheses will need to be tested that the detection of an active T cell response to CMV can function as a biomarker for predicting the efficacy of different therapeutic interventions or adverse effects, permitting improvement of therapeutic strategies, and personalizing them to individual IBD patients. 
